# Perforating scleral vessels adjacent to myopic choroidal neovascularization achieved a poor outcome after intravitreal anti-VEGF therapy

**DOI:** 10.3389/fmed.2022.1065397

**Published:** 2022-12-13

**Authors:** Wangjing Yao, Jiawen Xu, Xiangjun She, Jiangxin Yu, Zhi Liang, Xin Ye, Jiwei Tao, Sulan Wu, Jianbo Mao, Yiqi Chen, Yun Zhang, Lijun Shen

**Affiliations:** ^1^Department of Vitreous and Retinal Center, Affiliated Eye Hospital of Wenzhou Medical University, Hangzhou, China; ^2^Zhejiang Chinese Medical University Affiliated Jiaxing TCM Hospital, Jiaxing, China; ^3^Hwa Mei Hospital, University of Chinese Academy of Sciences (Ningbo No.2 Hospital), Ningbo, China; ^4^Zhejiang Provincial People's Hospital (Affiliated People's Hospital, Hangzhou Medical College), Hangzhou, China

**Keywords:** perforating scleral vessel, anti-VEGF therapy, optical coherence tomography, choroidal neovascularization, pathological myopia

## Abstract

**Background:**

This study aimed to summarize the features of perforating scleral vessels (PSVs) in patients with myopic choroidal neovascularization (CNV) (mCNV) using optical coherence tomography angiography (OCTA) and to identify the associations with the response after intravitreal anti-vascular endothelial growth factor (anti-VEGF) therapy.

**Methods:**

A consecutive series of naïve patients who had mCNV and received intravitreal anti-VEGF therapy with a follow-up duration of 12 months or more were enrolled. The prevalence, location, and branches of PSVs were analyzed. Projection-resolved OCTA (PR-OCTA) was used to analyze the neovascular signals between CNV and PSVs. Best corrected visual acuity (BCVA) and central macular thickness (CMT) were measured. The proportion of CMT change relative to baseline was used to assess therapeutic response.

**Results:**

A total of 44 eyes from 42 patients with mCNV were enrolled. PSVs were identified in 41 out of 44 eyes. Branches were identified in the PSVs of 24 eyes (57.14%), and 20 eyes did not have PSV branches (47.62%). In eight eyes (18.18%), PSVs were adjacent to mCNV, and in 36 eyes (81.82%), PSVs were not adjacent to mCNV. After anti-VEGF therapy for mCNV, BCVA increased (*F* = 6.119, *p* < 0.001) and CMT decreased (*F* = 7.664, *p* < 0.001). In the eyes where PSVs were adjacent to mCNV, BCVA improvements (*F* = 7.649, *p* = 0.009) were poor, and changes in CMT were small.

**Conclusion:**

The eyes with PSVs adjacent to mCNV showed poor therapeutic responses after intravitreal anti-VEGF therapy.

## Introduction

Pathologic myopia (PM) is defined by degenerative pathological changes in the retina and the choroid (1). Globally, PM is a major cause of blindness, especially in Eastern Asian countries (2–4). Among various macular complications, choroidal neovascularization (CNV) is the main reason for vision loss with a prevalence of 4–11% in patients with PM (5–8).

Choroidal ischemia is considered a major cause of CNV, and choroidal capillaries are considered to be the origin of myopic CNV (mCNV) (9). The choroidal thickness becomes thinner with the elongation of the axial length, resulting in atrophy of the choroid and the large choroidal vessels (10). A previous study highlighted that mCNV always developed around the atrophic regions (11) where the entire choroid might be absent. Under such conditions, choroidal capillaries are absent and therefore cannot be the origin of mCNV. Thus, the origin of CNV needs further study.

Querques et al. (12) stated that perforating scleral vessels (PSVs) were mainly detected in lacquer cracks and that they might contribute to the formation of myopic maculopathy. Ruiz-Medrano et al. (13) reported that PSVs were detected in 93.5% (145/155) of eyes with mCNV. Ohno-Matsui et al. (14) and Louzada et al. (15) reported that CNVs were connected to scleral blood vessels, and they supposed that the origin of CNV might be the intrascleral vessels. In Harvey et al.'s (16) latest study, scleral pits were described as superficial scleral excavations around the posterior ciliary arteries as a result of loss of the retinal pigment epithelium (RPE) and the destruction of Bruch's membrane. Through continuous OCT scanning, Sayanagi et al. (17) found a connection between PSVs and mCNV. Some were in direct contact, and others were connected *via* a thin choroid. Therefore, we inferred that PSVs may be involved in the pathology of mCNV.

Previous studies reported several cases of mCNV connected to PSVs; however, the clinical significance of PSVs in the pathology of mCNV remains unclear. Our study summarizes the features of PSVs and aims to identify their clinical significance.

## Methods

### Subjects and enrollment criteria

This was a retrospective observational study. We enrolled 272 eyes diagnosed with naïve mCNV. A consecutive series of 42 patients (44 eyes) who were seen at the Affiliated Eye Hospital of Wenzhou Medical University between 1 January 2018 and 30 August 2021 with a follow-up duration of at least 12 months were included in the final analysis. This study followed the tenets of the Declaration of Helsinki with respect to research involving human subjects. All patients were informed of the details of the research and agreed to sign a written general consent to participate in this observational study, which was approved by the ethics committee of the Affiliated Eye Hospital of Wenzhou Medical University.

The inclusion criteria were as follows: (1) age >18 years; (2) a refractive error (spherical equivalent) of ≤ -6.00 Diopter (D) or an axial length of ≥26.00 mm; and (3) a fundus showing degenerative changes in the sclera, the choroid, and the retina. The exclusion criteria were as follows: (1) CNV secondary to other ocular diseases (including vitreoretinal diseases, age-related macular degeneration, retinal vascular diseases, a history of central serous retinopathy, macular dystrophies, or inflammation); (2) poor image quality resulting from opacities of the media; (3) a history of intraocular surgery, including photodynamic therapy, vitrectomy, or intravitreal injection (IVI); (4) the quality of optical coherence tomography angiography (OCTA) scanning images lower than Q5; and (5) a follow-up duration of < 12 months.

All patients completed comprehensive examinations, including best corrected visual acuity (BCVA) using a standard logarithmic visual acuity chart, refractive error, axial length measurement (IOL Master; Carl Zeiss, Tubingen, Germany), structural spectral-domain optical coherence tomography (SD-OCT) (Spectralis SD-OCT; Heidelberg Engineering, Heidelberg, Germany), OCTA (Angio OCT; Optovue, Fremont, CA, USA), and swept-source-OCTA (SS-OCTA) (VG200D; SVision Imaging, Ltd., Luoyang, China), which are reported to work at near to 1,050 nm and feature a combination of industry-leading specifications including an ultrafast scan speed of 200,000 scans/s, a widefield of 56°, and an imaging depth of 2.7 mm (in tissue). Slit-lamp anterior examination, noncontact tonometry (TX-20, Canon, Tokyo, Japan), and indirect fundus ophthalmoscopy were also performed. If the patient met the criteria, both eyes underwent all the examinations.

Patients' clinical records were analyzed for basic data collection. All patients underwent 1+*pro-re-nata* (PRN) treatment and were monitored on a monthly basis. The type of medicine and the number of IVIs, the date of the first IVI, and the follow-up duration were recorded.

### Definition of PSVs

**Figure 1:**
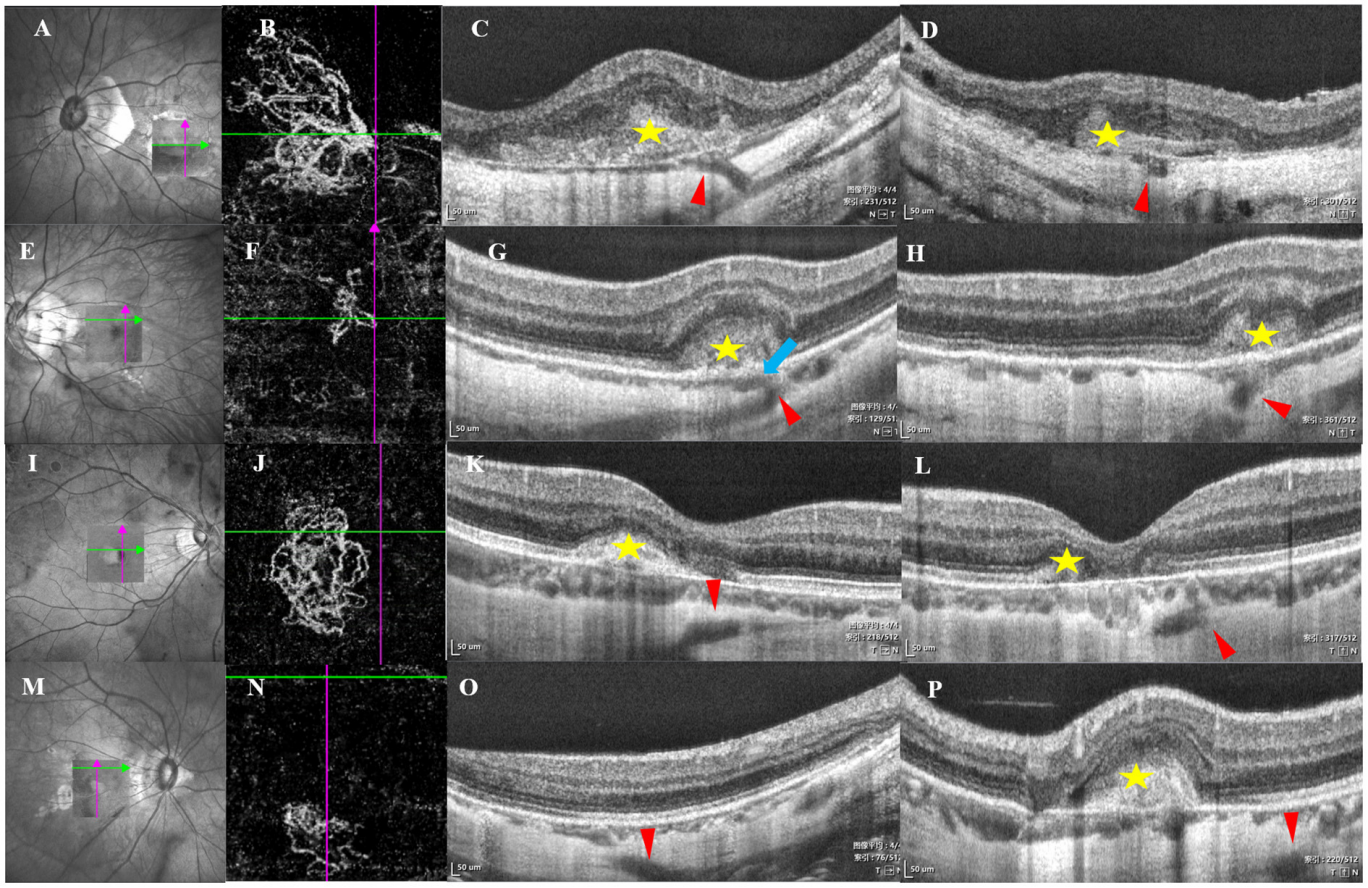
B-scan imaging of perforating scleral vessels (PSVs) with myopic choroidal neovascularization (mCNV) using swept-source-OCT (SS-OCT). The left arrow indicated the scanning laser ophthalmoscopy images, and the box indicated the scan area by SS-OCT. The second arrow indicated choroidal neovascularization (CNV) by optical coherence tomography angiography (OCTA), the cross lines indicated the location of PSV, and the right two arrows indicated horizontal and vertical B-scan images corresponding to the location of the left and second arrows. **(A–D)** The PSV adjacent to choroidal neovascularization (CNV), the red arrowhead indicated the PSV, which looked like an intrascleral low-reflection lumen-like and linear hyporeflective structure entering obliquely from the scleral regions, yellow star stands for mCNV, Bruch's membrane was defective and continuous with PSVs, a hollow hyporeflective space stands for PSVs at the vertical orientation corresponding to B, the hyporeflective structure was adjacent to CNV by defective Bruch's membrane. **(E–H)** PSVs penetrated into the dilated choroidal vessels under mCNV. The red arrowhead indicated the PSVs, a linear hyporeflective structure that protruded into the choroidal compartment and branched out to the enclosed and enlarged hyporeflective spaces, which indicated the dilated choroidal vessels (blue arrow). An enlarged hyporeflective space was detected in H, indicating PSVs, dilated choroidal space was near the PSVs. **(I–L)** The PSV (red arrowhead) passed under mCNV (yellow star). **(M–P)** The PSV (red arrowhead) was far away from mCNV (yellow star).

The images of 512 consecutive horizontal and vertical B-scans (3 × 3 mm) were acquired using the angio OCT (Optovue, Fremont, CA, USA) and analyzed by two retinal specialists. According to a previous study (14), a distinct sharp delineation of the choroid–scleral interfaces was first identified, and a linear hyporeflective structure that protruded obliquely from below the choroid–scleral interface was defined as a PSV (Figure 1). As the consecutive images of the B-scan were analyzed, the PSVs were judged by two retinal specialists. If two experts disagreed, a senior specialist would make the final decision.

### The morphology of PSVs related to CNV using OCT and OCTA

**Figure 2:**
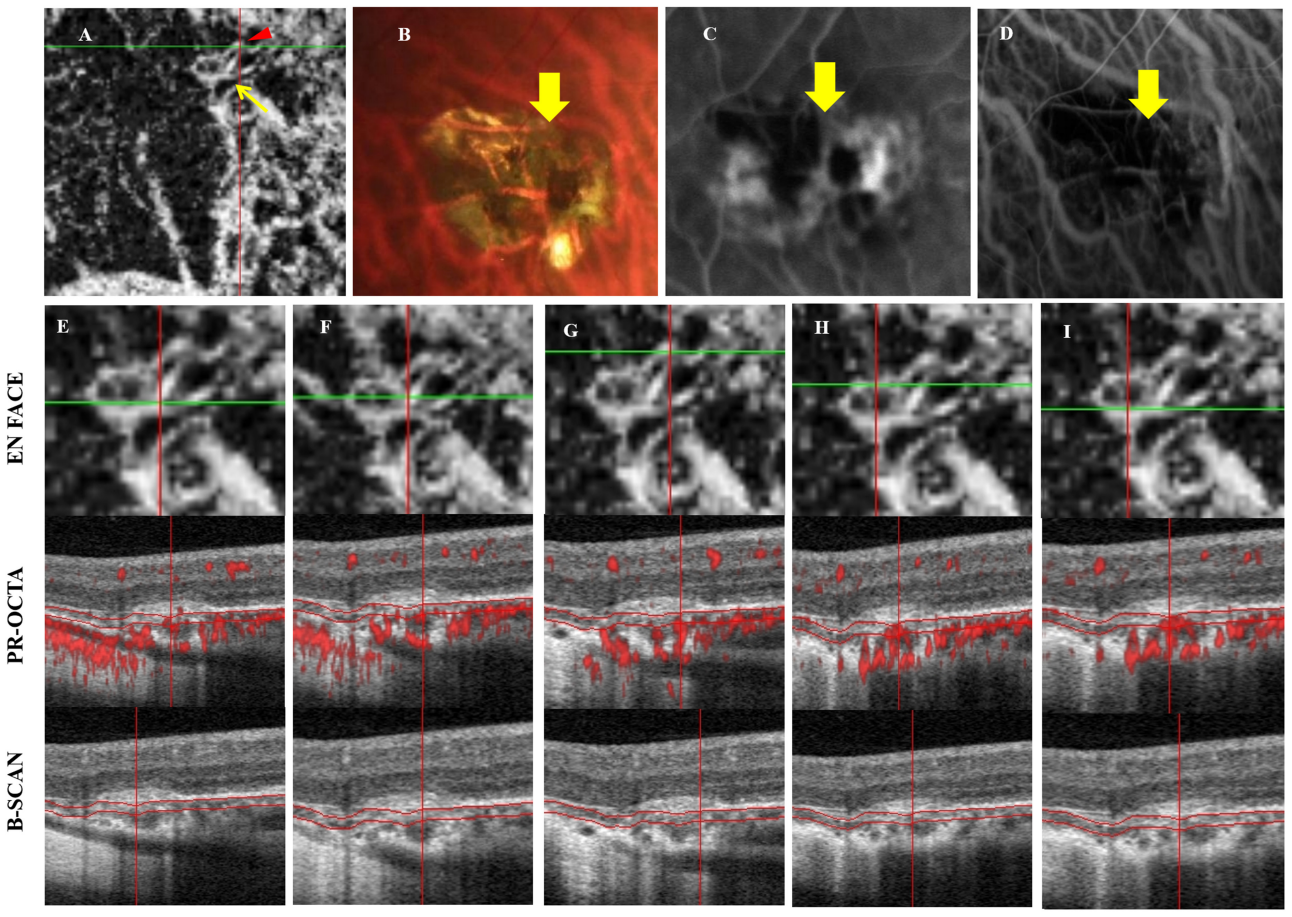
Multimodal images of PSV adjacent to mCNV. A 39-year-old man complained of vision loss in his right eye for 3 weeks, his refractive error was −16.50 D **(A)** OCTA indicated mCNV, yellow arrow indicated a ring-shaped CNV, where a possible feeder vessel is identified (red arrowhead). **(B)** Fundus photography showed an atrophic mCNV and hemorrhage, yellow arrow indicated the PSV corresponding to **(A)**; **(C,D)** The leakage of FFA and high fluorescence of ICGA corresponding to **(A)**. **(E**–**I)** The OCTA after using shadow removal in each scanning position. **(E,F)** Indicated the scanning positions of PSV adjacent to CNV, a hyporeflective linear region was detected below CNV, Bruch's membrane was defective in **(F)**, neovascularization signal was detected through PSVs and extended to the choroidal and Bruch's membrane. The low, reflective structure was adjacent to CNV. **(G)** The scanning position of PSV adjacent to CNV, and the neovascularization signal was detected through PSV and extended below Bruch's membrane. The “S”-shaped vessel may indicate that CNV may arise from PSVs and anastomoses with CNV. **(H,I)** indicated that the neovascularization signal of CNV without PSV originated from the choroid.

The eyes were divided into two groups based on the location of PSVs with CNV: (1) a group with PSVs adjacent to the CNV (a linear hyporeflective structure was directly adjacent to CNV *via* a defect in Bruch's membrane) (Figures 1A–D) and (2) a group with no PSVs adjacent to CNV. There were three manifestations in the second group: (a) PSVs penetrated into the dilated choroidal vessels under mCNV (a linear hyporeflective structure protruded into the choroidal compartment and branched out to the enclosed and enlarged hyporeflective spaces, which indicated the dilated choroidal vessels) (Figures 1E–H); (b) PSVs passed under mCNV (a linear hyporeflective structure penetrated below CNV without a connection) (Figures 1I–L); and (c) PSVs had no significant relationship with the position of mCNV (Figures 1M–P). The criterion for adjacency is whether there is a defect in Bruch's membrane in the retina at the junction of PSV and CNV.

In the OCTA 3 × 3 mm B-scans, branches of PSVs were detected as one or more low-reflection lumen-like structures on OCT images from the sclera to the choroid . PSVs were divided into the “with branches” group and the “without branches” group. If more than one low-reflection lumen-like structure was observed, the PSVs belonged to the with branches group (Supplementary Figure S1A). Otherwise, they belonged to the without branches group (Supplementary Figure S1B).

Optical coherence tomography angiography scans were used to acquire the entire CNV using commercial equipment (Angio OCT; Optovue). Neovascularization signals were analyzed after adopting projection-resolved OCT as previously introduced below Bruch's membrane (Figure 2) (18).

### Measurement of central macular thickness

The thinnest point of the central macular thickness (CMT) in the fovea was measured before and after treatment, which was the distance from the inner limiting membrane of the retina to the inner side of a strong reflection zone of the RPE. Each eye was measured three times by the same experienced examiner using a follow-up model with SD-OCT. The changes in CMT were defined as CMT at baseline minus CMT at 1, 3, 6, and 12 months. The reduction ratio was defined as the change in CMT divided by CMT at baseline. A previous study showed that a negative ratio or 0 indicated a poor response, 0–0.25 indicated a moderate response, and 0.25 or higher indicated a good response (19).

### Statistical analyses

All continuous variables were tested for normality using the Kolmogorov–Smirnov test. Normally distributed variables are expressed as mean ± standard deviation (SD) (*x* ± *s*), and non-normally distributed variables are expressed as the median (interquartile range) (*M*[*Q*]). The independent *t*-test or nonparametric test (the Mann–Whitney *U* test) was used to compare different groups as needed. An analysis of variance (ANOVA) and generalized estimating equation (GEE) were used to analyze the repeated data in normally distributed and non-normally distributed groups, respectively. The generalized linear mixed (GLM) model was used to explore the therapeutic response of PSVs. In the GLM model, a poor response was defined as 0, a moderate response was defined as 1, and a good response was defined as 2. A *P*-value < 0.05 indicated a statistically significant difference. All data analyses were performed using SPSS 25.0 (SPSS, Inc., Chicago, IL, USA).

## Results

### Demographic characteristics of participants

Of the 44 eyes of 42 patients with a follow-up duration of 12 months or more, there were 13 men and 29 women. The mean age was 54.66 ± 16.44 years (range, 22–85 years). The mean refractive error was −13.48 ± 4.08 D (range, −25 to −6 D). The median BCVA was 0.61 (0.30–1.00) logMAR at baseline (Table 1). After dividing the eyes into those with PSV with branches and those with PSV without branches, we had 24 eyes that had branches and 20 eyes that did not have branches. The mean age was 51.13 ± 16.33 years in the with branches group and 57.28 ± 16.32 years in the without branches group (*p* = 0.252). The mean refractive error was −13.43 ± 4.25 D in the with branches group and −13.72 ± 4.16 D in the without branches group (*p* = 0.805). The median BCVA at baseline was 0.70 (0.26–1.15) in the with branches group and 0.70 (0.46–1.15) in the without branches group (*p* = 0.315).

**Table 1:** Clinical characteristics of the eyes with choroidal neovascularization secondary to pathological myopia.

**Eyes with perorating scleral vessels**				**PSV with CNV**			
**Variables**	**Total**	**With branches**	**Without branches**	***P*-value**	**Adjacent to CNV**	**Not adjacent to CNV**	***P*-Value**
**Eyes (number)**	44	24	20		8	36	
**Age (y)**							
mean ± SD	54.66 ± 16.44	51.13 ± 16.33	57.28 ± 16.32	0.252	56.25 ± 14.42	53.24 ± 17.01	0.766
(range)	(22–85)	(22–85)	(26–74)		(39–80)	(22–85)	
**Refractive error (D)**							
mean ± SD	−13.48 ± 4.08	−13.43 ± 4.25	−13.72 ± 4.16	0.805	−12.77 ± 4.20	−13.65 ± 4.09	0.588
(range)	(−25 to −6)	(−25 to −6)	(−21.375 to −6.125)		(−17.88 to −6.00)	(−25.00 to −6.13)	
**BCVA**							
Median (interquartile range)	0.61 (0.30–1.00)	0.70 (0.26–1.15)	0.70 (0.46–1.15)	0.315	0.66 (0.41–1.06)	0.70 (0.35–1.15)	0.920
(range)	(0.097–1.85)	(0.097–1.85)	(0.30–1.30)		(0.22–1.30)	(0.097–1.85)	

CNV means choroidal neovascularization, BCVA means best corrected vision acuity, p < 0.05 was considered as significant, Mann-Whitney test.

In the PSV with branches group, branches were not adjacent to CNV in 36 eyes, and branches were adjacent to CNV in eight eyes. The mean age was 56.25 ± 14.42 years in the adjacent CNV group and 53.24 ± 17.01 years in the nonadjacent group (*p* = 0.766). The mean refractive error was −12.77 ± 4.20 D in the adjacent group and −13.65 ± 4.09 D in the nonadjacent group (*p* = 0.588). The median BCVA was 0.66 (0.41–1.06) in the adjacent group and 0.70 (0.35–1.15) in the nonadjacent group (*p* = 0.920). Subgroup analysis showed that 18.2% (8 out of 44) of PSVs were adjacent to CNV, 45.5% (22 out of 44) of PSVs were transverse to the thin choroid below mCNV, 9.09% (4 out of 44) of PSVs were below CNV, and 22.7% (10 out of 44) of PSVs had no significant correlation with the position of mCNV. Age (*p* = 0.418), refractive error (*p* = 0.319), and BCVA (*p* = 0.708) did not differ significantly between the four groups.

### BCVA improvements and the morphology of PSVs after intravitreal anti-vascular endothelial growth factor therapy

The 44 eyes of 42 patients were administered an intravitreal anti-vascular endothelial growth factor (anti-VEGF) drug (ranibizumab or conbercept) in a random regime (26 eyes received ranibizumab, 14 eyes received conbercept, and four eyes received a mixture of the two). There was no significant difference in drug selection between the different locations of PSVs (χ^2^ = 0.033, *p* = 0.875). In addition, we take both the drug types and the location of PSVs as covariables for repeated measurement and analysis. The effect of different drug types on BCVA is not significant (*F* = 0.092, *p* = 0.764), while the effect of the different locations of PSVs on BCVA is significant (*F* = 20.518, *p* < 0.001). Overall, BCVA improved from 0.61 (0.30–1.00) to 0.30 (0.22–0.52) at 1 month, to 0.30 (0.19–0.52) at 3 months, to 0.30 (0.15–0.52) at 6 months, and to 0.40 (0.19–0.61) at 12 months (*F* = 6.119, *p* < 0.001).

**Figure 3:**
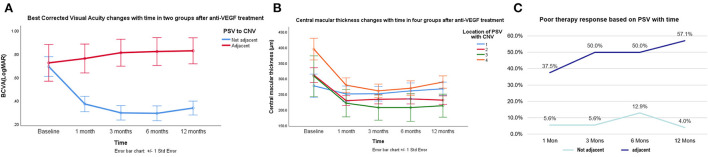
Changes of BCVA and CMT based on PSV after IVI at a follow-up time of 12 months. **(A)** BCVA was transformed into normalized data analysis by its natural logarithm. The PSV was classified into two categories, PSV adjacent to CNV or not, the horizontal ordinate represents time, the vertical ordinate represents BCVA. There was a significant difference between the two groups by analysis of variance (ANOVA) (*F* = 7.649, *p* = 0.009). **(B)** The changes in CMT were indicated in four types of PSV, 1 represents the trend of CMT change in the group PSVs adjacent to mCNV, 2 represents the trend of CMT change in the group PSV penetrated into the dilated choroidal vessels, 3 represents the trend of CMT change in the group PSVs passed under mCNV, and 4 represents the trend of CMT change in the group PSVs, which had no significant correlation with the position of mCNV; there was no significant difference between groups by the generalized estimating equation (GEE). **(C)** Poor therapeutic response based on the PSV with time, the PSV was divided into those adjacent to CNV and those not; there was a significant difference between the two groups, and the generalized linear mixed (GLM) model predicts that PSV adjacent to CNV is less likely to get a good response than PSV not adjacent to CNV.

Best corrected visual acuity in the eyes with PSVs adjacent to CNV improved from 0.66 (0.41–1.06) to 0.52 (0.41–1.15) at 1 month, to 0.52 (0.46–1.30) at 3 months, to 0.75 (0.41–1.20) at 6 months, and to 0.85 (0.46–1.20) at 12 months. BCVA in the eyes with PSVs not adjacent to CNV improved from 0.70 (0.3–1.08) to 0.30 (0.14–0.57) at 1 month, to 0.22 (0.15–0.40) at 3 months, to 0.22 (0.14–0.40) at 6 months, and to 0.40 (0.10–0.52) at 12 months. There was a significant difference between the two groups in terms of time (*F* = 7.649, *p* = 0.009). In subgroup analysis, BCVA in eyes with PSVs that penetrated into the dilated choroidal vessels under mCNV improved from 0.70 (0.30–1.00) to 0.30 (0.10–0.52) at 1 month, to 0.22 (0.15–0.40) at 3 months, to 0.22 (0.10–0.40) at 6 months, and to 0.40 (0.10–0.52) at 12 months. BCVA in eyes with PSVs under CNV improved from 0.40 (0.35–0.55) to 0.30 (0.26–0.30) at 1 month, to 0.22 (0.22–0.26) at 3 months, to 0.22 (0.19–0.26) at 6 months, and to 0.22 (0.16–0.26) at 12 months. BCVA in the eyes with PSVs not related to CNV improved from 0.66 (0.41–1.15) to 0.30 (0.13–0.52) at 1 month, to 0.26 (0.13–0.35) at 3 months, to 0.22 (0.15–0.35) at 6 months, and to 0.22 (0.13–0.40) at 12 months. There was a significant difference in BCVA changes between the eyes with PSVs adjacent to CNV and the eyes without PSVs adjacent to CNV (*F* = 2.982, *p* = 0.048) (Table 2; Figure 3A). The eyes with PSVs that were not adjacent to CNV tended to show greater improvement in BCVA than the eyes with PSVs adjacent to CNV.

**Table 2:** The changes of BCVA and central macular thickness after Anti-VEGF therapy.

**Variables**		**BCVA**							**CMT(**μ**m)**						
		**Baseline**	**1M**	**3M**	**6M**	**12M**	**F**	***P* Value**	**Baseline**	**1M**	**3M**	**6M**	**12M**	**F**	***P* Value**
**Eyes with PSV**	**With Branches**	0.70 (0.22–1.30)	0.40 (0.10–0.75)	0.26 (0.15–0.76)	0.30 (0.10–0.76)	0.40 (0.16–1.00)	6.083	< 0.001^a^	284.0 (220.0–348.0)	227.0 (202.0–257.0)	229.0 (189.0–270.0)	226.0 (194.0–260.0)	250.0 (196.0–273.0)	8.697	< 0.001^c^
	**Without Branches**	0.52 (0.40–0.80)	0.30 (0.26–0.52)	0.30 (0.22–0.46)	0.26 (0.19–0.46)	0.26 (0.19–0.46)	1.164	0.289^b^	389.0 (282.0–428.0)	230.0 (204.0–284.0)	235.0 (204.0–287.0)	282.0 (208.0–307.0)	261.0 (218.0–276.0)	1.739	0.198^d^
**PSV with CNV**	**Adjacent to CNV**	0.66 (0.41–1.06)	0.52 (0.41–1.15)	0.52 (0.46–1.30)	0.75 (0.41–1.20)	0.85 (0.46–1.20)	5.788	0.002^a^	281.0 (268.0–296.0)	244.0 (210.0–294.0)	251.5 (231.0–279.0)	272.5 (226.0–282.0)	272.5 (218.0–311.0)	5.178	0.004^c^
	**Penetrated into DCV**	0.70 (0.30–1.00)	0.30 (0.10–0.52)	0.22 (0.15–0.40)	0.22 (0.10–0.40)	0.40 (0.10–0.52)	2.982	0.048^b^	299.0 (224.0–363.5)	227.0 (190.5–253.0)	219.0 (192.5–277.0)	229.0 (192.0–272.0)	227.0 (194.5–261.0)	1.344	0.282^d^
	**Under CNV**	0.40 (0.35–0.55)	0.30 (0.26–0.30)	0.22 (0.22–0.26)	0.22 (0.19–0.26)	0.22 (0.16–0.26)			308.0 (227.0–389.0)	221.0 (196.0–246.0)	207.0 (168.0–246.0)	207.0 (168.0–246.0)	213.0 (168.0–258.0)		
	**Not related to CNV**	0.66 (0.41–1.15)	0.30 (0.13–0.52)	0.26 (0.13–0.35)	0.22 (0.15–0.35)	0.22 (0.13–0.40)			395.0 (369.5–438.0)	230.0 (205.0–337.0)	206.0 (203.5–320.0)	208.0 (203.5–315.5)	276.0 (238.5–331.5)		

BCVA means best corrected vision acuity, PSV means perforating scleral vessel, CMT means central macular thickness, CNV means choroid neovascularization, DCV means dilated choroidal vessels, p < 0.05 was considered as significant, by Repeated Measures ANONA or GEE. P

a stands for VA changes with time by ANONA analysis, P

b stands for VA changes in different groups interacted with time by ANONA analysis, P

c stands for CMT changes with time by GEE, P

d stands for CMT changes in different groups interacted with time by GEE. GEE means generalized estimating equation; ANONA means analysis of Variance.

In the PSV without branches group, BCVA improved from 0.52 (0.40–0.80) to 0.30 (0.26–0.52) at 1 month, to 0.30 (0.22–0.46) at 3 months, to 0.26 (0.19–0.46) at 6 months, and to 0.26 (0.19–0.46) at 12 months (*p* < 0.001). In the PSV with branches group, VA improved from 0.70 (0.22–1.30) to 0.40 (0.10–0.75) at 1 month, to 0.26 (0.15–0.76) at 3 months, to 0.30 (0.10–0.76) at 6 months, and to 0.40 (0.16–1.00) at 12 months (*p* < 0.001). There was no significant difference between the with branches and without branches groups in terms of time (*F* = 1.164, *p* = 0.289) (Table 2).

### Patients with PSVs adjacent to mCNV achieved poor outcomes

Central macular thickness decreased from 301.5 (228.0–389.0) to 228.0 (202.0–284.0) μm at 1 month, to 230.0 (199.0–284.0) μm at 3 months, to 230.0 (199.0–284.0) μm at 6 months, and to 259.5 (204.0–276.0) μm at 12 months after therapy (*F* = 7.664, *p* < 0.001). CMT in the eyes with PSVs without branches decreased from 389.0 (282.0–428.0) μm to 230.0 (204.0–284.0) μm at 1 month, to 235.0 (204.0–287.0) μm at 3 months, to 282.0 (208.0–307.0) μm at 6 months, and to 261.0 (218.0–276.0) μm at 12 months. CMT in the eyes with PSVs with branches decreased from 284.0 (220.0–348.0) μm to 227.0 (202.0–257.0) μm at 1 month, to 229.0 (189.0–270.0) μm at 3 months, to 226.0 (194.0–260.0) μm at 6 months, and to 250.0 (196.0–273.0) μm at 12 months. There were no significant differences between the groups (*F* = 1.739, *p* = 0.198).

Central macular thickness in the eyes with PSVs adjacent to CNV decreased from 281.0 (268.0–296.0) μm to 244.0 (210.0–294.0) μm at 1 month, to 251.5 (231.0–279.0) μm at 3 months, to 272.5 (226.0–282.0) μm at 6 months, and to 272.5 (218.0–311.0) μm at 12 months. CMT in eyes with PSVs that penetrated into the choroid under CNV decreased from 299.0 (224.0–363.5) μm to 227.0 (190.5–253.0) μm at 1 month, to 219.0 (192.5–277.0) μm at 3 months, to 229.0 (192.0–272.0) μm at 6 months, and to 227.0 (194.5–261.0) μm at 12 months. CMT in the eyes with PSVs under CNV decreased from 308.0 (227.0–389.0) to 221.0 (196.0–246.0) μm at 1 month, to 207.0 (168.0–246.0) μm at 3 months, to 207.0 (168.0–246.0) μm at 6 months, and to 213.0 (168.0–258.0) μm at 12 months. CMT in the eyes with PSVs not related to CNV decreased from 395.0 (369.5–438.0) to 230.0 (205.0–337.0) μm at 1 month, to 206.0 (203.5–320.0) μm at 3 months, to 208.0 (203.5–315.5) μm at 6 months, and to 276.0 (238.5–331.5) μm at 12 months (Table 2). There was no significant difference between groups (*F* = 1.344, *p* = 0.282) (Figure 3B).

**Figure 4:**
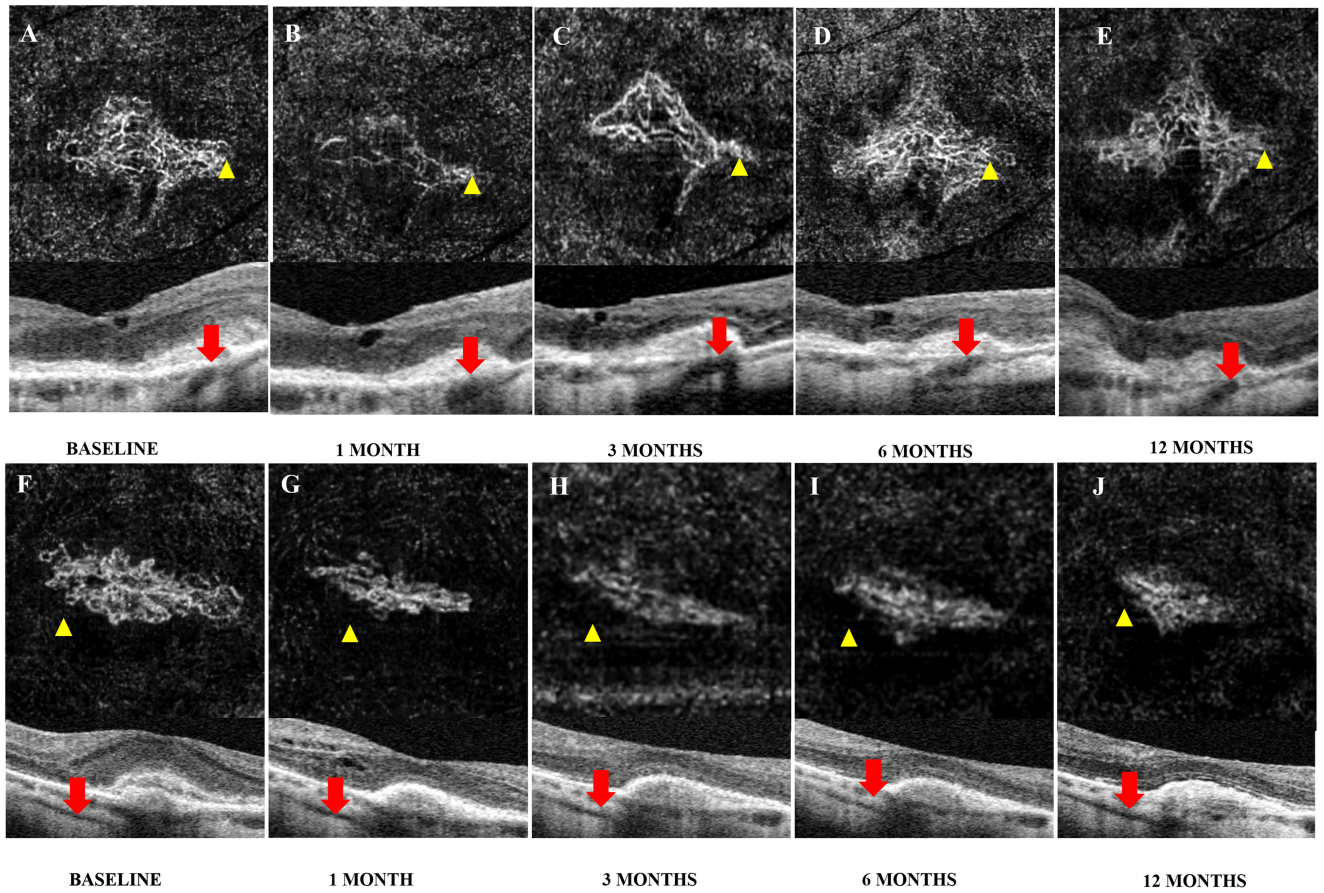
Images of changes in CNV adjacent to PSVs or not by OCTA after intravitreal injection (IVI). A 64-year-old woman complained of vision loss in her right eye for 15 days. The baseline best corrected visual acuity (BCVA) was 0.80 (20/125), and the central macular thickness (CMT) was 278 μm; she received four ranibizumab injections (initial injection, 1, 7, and 18 months after the initial treatment) during the follow-up period. **(A–C)** The size of both CNV and CMT decreased at 1 month after the initial injection and increased slightly at 3 months. **(D)** CNV recurred 6 months after the initial injection, and with an increase in the size of both CNV and CMT, one additional injection of ranibizumab was given at 7 months. **(E)** The size of CNV at 12 months was slightly smaller than that at 6 months. A 66-year-old woman complained of vision loss in her left eye for 10 days; BCVA at baseline was 1.00 (20/200) and CMT was 395 μm. The patient received two conbercept injections (initial injection and 2 months after the initial treatment) during the follow-up period. **(F–J)** The size of both CNV and CMT decreased at 1 month after the initial injection and remained stable for the following year.

The therapeutic response was defined as the changes in CMT divided by CMT at baseline. The results revealed that 31.8% of the eyes showed a good response, 56.8% showed a moderate response, and 11.4% showed a poor response at 1 month after therapy. At 3 months, 34.1% of the eyes showed a good response, 52.3% showed a moderate response, and 13.6% showed a poor response. At 6 months, 37.8% of the eyes showed a good response, 43.2% showed a moderate response, and 18.9% showed a poor response. At 12 months, 31.3% of the eyes showed a good response, 53.1% showed a moderate response, and 15.6% showed a poor response. After the GLM analysis, we found that the eyes with PSVs that were not adjacent to CNV were more likely to show a good response than the eyes with PSVs adjacent to CNV at 1 month [odds ratio (OR) = 0.080, *p* = 0.009], at 3 months (OR = 0.057, *p* = 0.004), at 6 months (OR = 0.111, *p* = 0.016), and at 12 months (OR = 0.029, *p* = 0.005) after therapy. The eyes with PSVs adjacent to CNV were more likely to show a poor therapeutic response (Figures 3C, 4; Table 3; Supplementary Tables S1–S3).

**Table 3:** Risk of therapy response at 12 months after intravitreal anti-VEGF and risk ratios associated factors.

**Characteristics**	**Percentage**	**Changes of CMT at 12M(μm)**	**Multivariable model**		
			**Risk ratio**	**95%confidence interval**	***P*-Value**
**Therapy Response**					
Good Response	31.3%	136.0(103.3–164.3)	0.012	0.001–0.160	0.001
Moderate Response	53.1%	35.0(22.0–53.0)	0.562	0.117–2.694	0.471
Poor Response	15.6%	−15.0(−50.0 to −15.0)	Reference		
**PSV with CNV**	**Good response(%)**				
PSV Adjacent to CNV	0%	0.0(−32.5–31.0)	Reference		
PSV not Adjacent to CNV	40%	59.0(23.0–119.0)	0.029	0.003–0.340	0.005
**Morphology of PSV**					
PSV With Branches	23.5%	29.0(4.5–71.0)	Reference		
PSV Without Branches	40%	69.5(30.3–116.5)	0.431	0.102–1.819	0.252

PSV means perforating scleral vessels, CMT means central macular thickness, CNV means choroidal neovascularization, p < 0.05 was considered as significant, using Generalized Linear Models.

## Discussion

Perforating scleral vessels are always detected in patients with mCNV. The loss of RPE and the disruption of Bruch's membrane are always detected at the position of scleral excavations around PSVs (16). Our study found that the eyes with PSVs adjacent to CNV showed poor outcomes after intravitreal anti-VEGF therapy.

Querques et al. (12) reported that the PSVs were detected in 75% of patients with mCNV. Louzada et al. (15) found that CNV was continuous with intrascleral blood vessels. Our results indicated that PSVs were detected in 41 of 44 eyes, and only eight eyes had PSVs adjacent to CNV through a defect in the RPE and Bruch's membrane. PSVs adjacent to mCNV are rarely seen. Even in a large-sample study, only 10 of more than 100 PSVs were connected to mCNV, and most of the PSVs seemed to transverse the thin choroid below mCNV. The authors suggested that scleral vessels may be the feeding vessels of mCNV (12).

Age, refractive error, and BCVA were not significantly different according to the location of PSVs. However, CMT in PSVs with no relationship to CNV was higher than that in the other groups (Figure 3B; Table 2). Research by Querques et al. (12) demonstrated that scleral expansion at the site of the PSVs might join to form lacquer cracks. Moreover, PSVs might increase the mechanical stress on Bruch's membrane (locus minoris resistentiae) (20). Another study showed that the scleral vessel might act as a tether or fixation that could concentrate forces and transmit forces to the choroid when the eye moves (12). We detected PSVs due to the loss of RPE and disruption of Bruch's membrane. Scleral pits could enlarge and could be mistaken to be the choroid with time (16). However, not all of the neovascularization signal of mCNV was from the choroid (Figure 2). Our study indicated that it might arise from the PSVs and anastomoses with choroidal CNV. As the reason was not clear, we inferred that local atrophy of Bruch's membrane and the choroid occurred early at the location of PSVs. With the degeneration of the choroid, neovascularization may arise from the scleral vessels adjacent to CNV.

After therapy, a good response was achieved in the eyes with PSVs not related to mCNV. On the other hand, a poor response was more likely in the eyes with PSVs adjacent to CNV (Figure 3C). Choroidal atrophy was consistently detected even when the choroid was nearly absent when PSVs were adjacent to CNV (Figures 1C,D, 4A–E). An ischemic choroid may be the reason for the poor response. Moreover, previous findings suggested that, if mCNV originates in the sclera, then the vessels may be more arterialized (21). Therefore, the response was poor. We also found another type of PSV that was adjacent to an enlarged choroid under CNV (Figures 1E–H). The dilated choroidal spaces were identified as dilated subfoveal choroidal veins (DCVs), which were reported in about 30% of mCNV cases, and some studies suggested that the dilated choroid represented an ischemic or inflammatory condition.

Perforating scleral vessels are considered the vascular origin of CNV and the efferent venules and DCVs (22). Whether this type of PSV that is adjacent to the choroid represents the final stage of mCNV is worth studying, as when the choroid becomes atrophied, the PSV may directly connect with mCNV at the final stage. More studies are needed to address this question.

**Figure 5:**
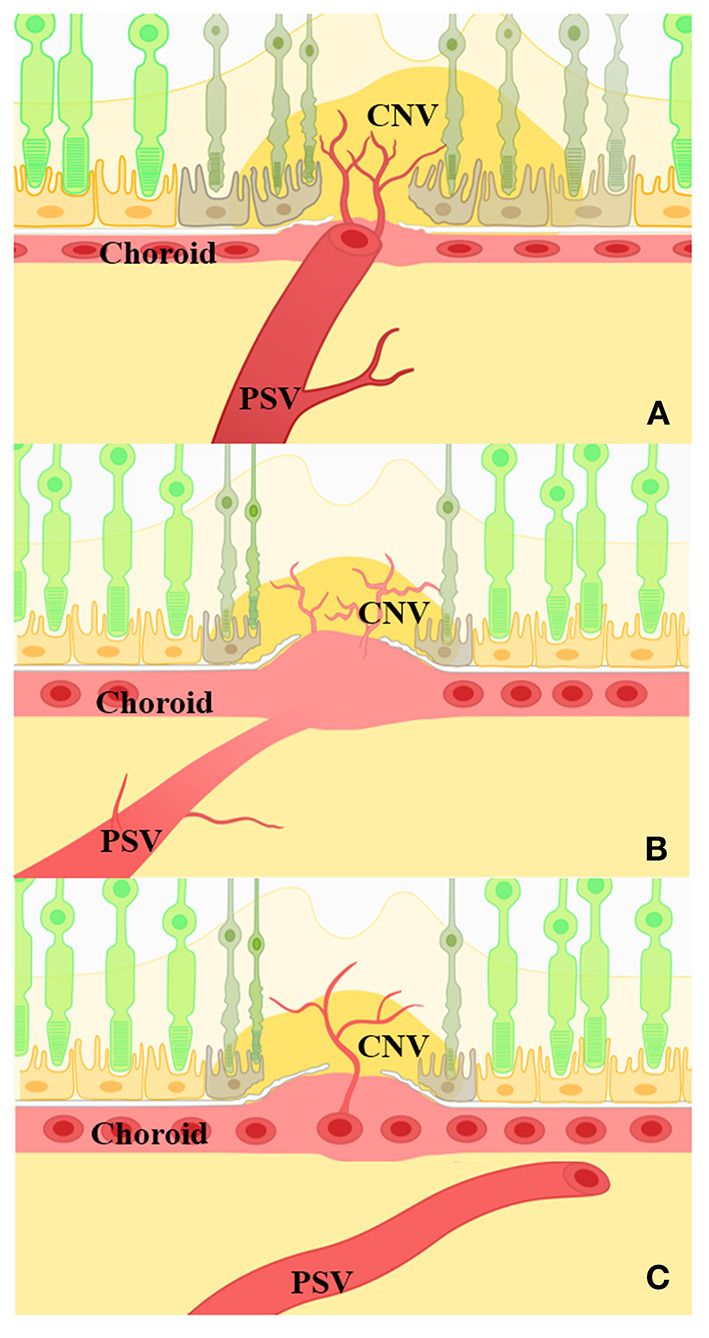
Schematic drawing of the possible relationship between PSVs and mCNV. **(A)** PSVs adjacent to mCNV *via* a defective Bruch's membrane. The choroid is thin or absent in this region. Photoreceptor and retinal pigment epithelium (RPE) cells were destroyed, and CNV may anastomose with PSVs. **(B)** PSVs penetrated into the dilated choroid; the dilated choroid was then adjacent to mCNV *via* a defective Bruch's membrane. The enlarged vessel under CNV indicated a dilated choroidal structure, and CNV may arise from the defective Bruch's membrane near the dilated choroidal vessels. **(C)** PSVs passed under mCNV. PSVs showed no significant correlation with the position of CNV.

Our research indicated that the eyes with PSVs adjacent to CNV achieved poor therapy results. This type of mCNV may arise from PSVs and result in anastomoses with choroidal neovascularization. Based on our findings, a schematic drawing of the possible relationship between PSVs and mCNV is shown in Figure 5. We hypothesized that, as PM progressed, the RPE, Bruch's membrane, and the choroid were destroyed, causing the adjacent PSVs to enlarge. When the outer border of the choroid was destroyed, PSVs adjacent to the dilated choroidal vessel were destroyed, the choroid became atrophied, and Bruch's membrane was destroyed. The origins of mCNV may be both PSVs and the choroid, and this explains the reason for poor outcomes.

We acknowledge several limitations of this study. First, the study was a retrospective study, and the sample size was small; therefore, a well-designed prospective study with a large sample size is needed to prove that the origin of mCNV is both the PSVs and the choroid. Second, no histopathologic assessment of clinical samples was undertaken in terms of the choroidal and scleral structure based on OCT. Third, the types of drugs used for IVI of anti-VEGF were different, and this may have an impact on the therapeutic effect. Fourth, fluorescein angiography and indocyanine green angiography should be used to further delineate the location of veins or arteries with PSVs and CNV and to learn about their relationships with PSV.

## Conclusions

In conclusion, eyes with PSVs adjacent to mCNV showed worse outcomes, but whether this represented the final stage of mCNV or was a coincidence remains unknown. The location of the PSVs with CNV needs to be considered in clinical practice, and the reasons for the differences associated with the location of PSVs need further research.

## Data availability statement

The raw data supporting the conclusions of this article will be made available by the authors, without undue reservation.

## Ethics statement

The studies involving human participants were reviewed and approved by the Ethics Committee of Eye Hospital of Wenzhou Medical University, Zhejiang Province, China. Written informed consent for participation was not required for this study in accordance with the national legislation and the institutional requirements.

## Author contributions

Study conception and design: XS, WY, JX, and LS. Data collection and analysis: WY, JX, JY, XY, JT, and ZL. Drafting the manuscript: WY, JX, and XS. Critically revising the manuscript for intellectual content: SW, JM, YC, YZ, and LS. All authors have read and approved the final manuscript.

## Supplementary material

Supplementary Figure S1 Branches of perforating scleral vessels (PSVs) judged by optical coherence tomography (OCT) images from the sclera to choroid in the 3 mm × 3 mm B-scan. **(A)** A low-reflection lumen-like structure was divided into two parts (white arrowheads), red arrow stands for myopic choroidal neovascularization (mCNV). **(B)** Only one low-reflection lumen-like structure (white arrowheads) was observed in the whole B-scan images.

Supplementary Table 1 Risk of therapy response at 1 month after intravitreal anti-VEGF therapy and risk ratios associated factors.

Supplementary Table 2 Risk of therapy response at 3 months after intravitreal anti-VEGF therapy and risk ratios associated factors.

Supplementary Table 3 Risk of therapy response at 6 months after intravitreal anti-VEGF therapy and risk ratios associated factors.
