# Supplementary figures and images for: Perforating scleral vessels adjacent to myopic choroidal neovascularization achieved a poor outcome after intravitreal anti-VEGF therapy
Source: Front Med (Lausanne). 2022 Dec 13;9:1065397. doi: 10.3389/fmed.2022.1065397 (PMC9792597; doi:10.3389/fmed.2022.1065397)

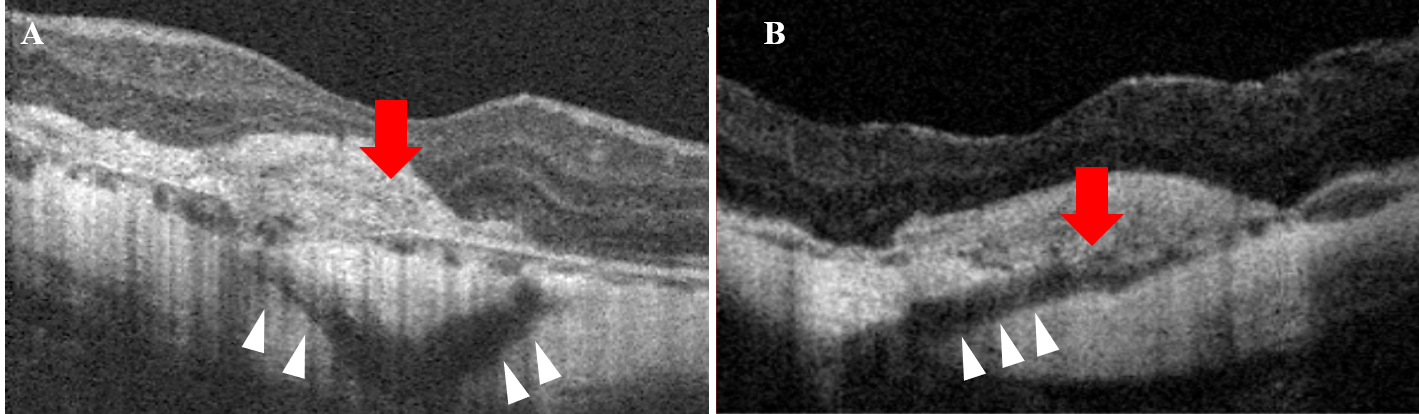

Supplement: Supplementary Figure S1 — Branches of perforating scleral vessels (PSVs) judged by optical coherence tomography (OCT) images from the sclera to choroid in the 3 mm × 3 mm B-scan. (A) A low-reflection lumen-like structure was divided into two parts (white arrowheads), red arrow stands for myopic choroidal neovascularization (mCNV). (B) Only one low-reflection lumen-like structure (white arrowheads) was observed in the whole B-scan images. [file Image_1.TIF]
