# Supplementary material for: Perforating scleral vessels adjacent to myopic choroidal neovascularization achieved a poor outcome after intravitreal anti-VEGF therapy
Source: Front Med (Lausanne). 2022 Dec 13;9:1065397. doi: 10.3389/fmed.2022.1065397 (PMC9792597; doi:10.3389/fmed.2022.1065397)
Supplement: Supplementary Table 1 — Risk of therapy response at 1 month after intravitreal anti-VEGF therapy and risk ratios associated factors. [file Table_1.DOCX]

| **STable-1 Risk of Therapy Response at 1 Month after intravitreal anti-VEGF therapy and Risk Ratios Associated Factors** | | | | | |
| --- | --- | --- | --- | --- | --- |
|  |  |  | **Multivariable Model** | | |
| **Characteristics** | **Percentage** | **Changes of CMT at 1M(μm)** | **Risk Ratio** | **95%confidence Interval** | **P Value** |
| **Therapy Response** |  |  |  |  |  |
| Good Response | 31.8% | 147.0(119.8 - 216.0) | 0.038 | 0.006-0.259 | 0.038 |
| Moderate Response | 56.8% | 32.0(22.0 - 53.0) | 1.310 | 0.327-5.237 | 0.703 |
| Poor Response | 11.4% | -6.0(-15.0 - 0.0) | Reference |  |  |
| **PSV with CNV** | **Good response（%）** | |  |  |  |
| PSV Adjacent to CNV | 0% | 20.0(-1.5 - 54.3) | Reference |  |  |
| PSV not Adjacent to CNV | 38.9% | 54.0(24.5 - 143.3) | 0.080 | 0.012-0.536 | 0.009 |
| **Morphology of PSV** |  |  |  |  |  |
| PSV With Branches | 26.1% | 34.0(11.5 - 92.8) | Reference |  |  |
| PSV Without Branches | 38.1% | 58.5(30.5 - 143.3) | 0.602 | 0.178-2.035 | 0.602 |
| PSV means perforating scleral vessels, CMT means central macular thickness, CNV means choroidal neovascularization, p <0.05 was considered as significant, using Generalized Linear Models. | | | | | |
