# Supplementary material for: Perforating scleral vessels adjacent to myopic choroidal neovascularization achieved a poor outcome after intravitreal anti-VEGF therapy
Source: Front Med (Lausanne). 2022 Dec 13;9:1065397. doi: 10.3389/fmed.2022.1065397 (PMC9792597; doi:10.3389/fmed.2022.1065397)
Supplement: Supplementary Table 2 — Risk of therapy response at 3 months after intravitreal anti-VEGF therapy and risk ratios associated factors. [file Table_2.DOCX]

| **STable-2 Risk of Therapy Response at 3 Months after intravitreal anti-VEGF therapy and Risk Ratios Associated Factors** | | | | | |
| --- | --- | --- | --- | --- | --- |
|  |  |  | **Multivariable Model** | | |
| **Characteristics** | **Percentage** | **Changes of CMT at 3M(μm)** | **Risk Ratio** | **95%confidence Interval** | **P Value** |
| **Therapy Response** |  |  |  |  |  |
| Good Response | 34.1% | 161.0(119.5 - 237.0) | 0.023 | 0.003-0.168 | <0.001 |
| Moderate Response | 52.3% | 32.0(20.0 - 52.0) | 0.722 | 0.177-2.943 | 0.649 |
| Poor Response | 13.6% | -20.5(-42.5 - -15.8) | Reference |  |  |
| **PSV with CNV** | **Good response（%）** | |  |  |  |
| PSV Adjacent to CNV | 0% | 10.5(-15.8 - 45.0) | Reference |  |  |
| PSV not Adjacent to CNV | 41.7% | 75.5(22.8 - 141.5) | 0.057 | 0.008-0.394 | 0.004 |
| **Morphology of PSV** |  |  |  |  |  |
| PSV With Branches | 26.1% | 33.5(6.0 - 83.5) | Reference |  |  |
| PSV Without Branches | 42.6% | 80.0(27.3 - 141.5) | 0.368 | 0.106-1.274 | 0.115 |
| PSV means perforating scleral vessels, CMT means central macular thickness, CNV means choroidal neovascularization, p <0.05 was considered as significant, using Generalized Linear Models. | | | | | |
