# Supplementary material for: Perforating scleral vessels adjacent to myopic choroidal neovascularization achieved a poor outcome after intravitreal anti-VEGF therapy
Source: Front Med (Lausanne). 2022 Dec 13;9:1065397. doi: 10.3389/fmed.2022.1065397 (PMC9792597; doi:10.3389/fmed.2022.1065397)
Supplement: Supplementary Table 3 — Risk of therapy response at 6 months after intravitreal anti-VEGF therapy and risk ratios associated factors. [file Table_3.DOCX]

| **STable-3 Risk of Therapy Response at 6 Months after intravitreal anti-VEGF therapy and Risk Ratios Associated Factors** | | | | | |
| --- | --- | --- | --- | --- | --- |
|  |  |  | **Multivariable Model** | | |
| **Characteristics** | **Percentage** | **Changes of CMT at 6M(μm)** | **Risk Ratio** | **95%confidence Interval** | **P Value** |
| **Therapy Response** |  |  |  |  |  |
| Good Response | 37.8% | 141.5(102.5 - 220.3) | 0.085 | 0.014-0.515 | 0.007 |
| Moderate Response | 43.2% | 39.5(23.0 - 60.0) | 0.833 | 0.175-3.969 | 0.819 |
| Poor Response | 18.9% | -23.0(-41.0 - -9.5) | Reference |  |  |
| **PSV with CNV** | **Good response（%）** | |  |  |  |
| PSV Adjacent to CNV | 0% | 3.0(-3.0 - 41.3) | Reference |  |  |
| PSV not Adjacent to CNV | 45.2% | 66.0(26.0 - 139.5) | 0.111 | 0.018-0.664 | 0.016 |
| **Morphology of PSV** |  |  |  |  |  |
| PSV With Branches | 31.6% | 48.0(18.0 - 109.8) | Reference |  |  |
| PSV Without Branches | 44.4% | 66.0(29.0 - 110.0) | 0.936 | 0.269-3.255 | 0.917 |
| PSV means perforating scleral vessels, CMT means central macular thickness, CNV means choroidal neovascularization, p <0.05 was considered as significant, using Generalized Linear Models. | | | | | |
